# Correlation of hyperglycemia and balthazar classification in patients with acute pancreatitis

**DOI:** 10.12669/pjms.40.10.6687

**Published:** 2024-11

**Authors:** Guner Kilic, Gulce Ecem Kilic, Adnan Ozkahraman, Yusuf Kayar

**Affiliations:** 1 Guner Kilic, Gastroenterology, Department of Internal Medicine, Van Training and Research Hospital, Van, Turkey; 2 Gulce Ecem Kilic, Internal Medicine, Department of Internal Medicine, Van Training and Research Hospital, Van, Turkey; 3 Adnan Ozkahraman, Internal Medicine, Department of Internal Medicine, Van Training and Research Hospital, Van, Turkey; 4 Yusuf Kayar, Gastroenterology, Department of Internal Medicine, Van Training and Research Hospital, Van, Turkey

**Keywords:** Acute pancreatitis, Hyperglycemia, Balthazar Classification

## Abstract

**Objective:**

High levels of glucose during acute pancreatitis (AP) progression influence disease progression by promoting the release of inflammatory cytokines. The aim of this study was to evaluate the effects of both the blood glucose level in patients without diabetes mellitus (DM) and the presence of DM on the severity and course of AP in patients presenting with clinical AP.

**Methods:**

The study included 343 patients who were hospitalized at Van Training and Research Hospital, Turkey and followed up with the diagnosis of AP between 2014 and 2018. The patients were separated into two groups as diabetic and non-diabetic. The relationship between DM and the severity and course of AP was examined in the two groups.

**Results:**

The DM group included 52 (15.1%) patients, and the non- DM group included 291 (84.9%) patients. In the non-DM group, the serum glucose level was <125 mg/dl in 160 (54.9 %) patients, and >125 mg/dl in 131 (45.1 %) patients. In the comparison of AP severity in the diabetic and non-diabetic groups, the rate of severe AP was determined to be significantly higher in the diabetic group according to the Modified Balthazar classification, evaluated from tomographies taken on admission and on the 3rd day (p:0.026, p:0.001, respectively).

**Conclusion:**

Elevated blood glucose is relatively common in patients with AP and has a negative impact on the disease process. A high glucose level can increase the severity of AP and slow healing.

## INTRODUCTION

Acute pancreatitis (AP) is an acute inflammatory disease of the pancreas. The clinical course of AP ranges from self-limiting mild pancreatitis to severe pancreatitis, which can lead to death.^1^,^2^ Many parameters are used to determine the severity of AP. Despite certain limitations, the imaging modality developed by Balthazar, which is used to evaluate pancreatic and peripancreatic inflammation and pancreatic necrosis, also plays an important role in determining the cause, diagnosis, staging, and complications of AP.^3^ There has also been shown to be a significant correlation between the levels of parameters such as glucose, creatinine, pancreatic enzymes, calcium, proinflammatory cytokines and C-reactive protein (CRP) and the severity of AP.^4^-^6^

In addition to all these, some host-related factors have been reported to affect the severity of AP.^7^ The coexistence of AP and diabetes mellitus (DM) has been known for many years.^8^ Previous studies have shown that just as diabetes increases the risk of AP 1.5-3-fold, this risk can also be reduced by blood sugar regulation.^9^

High levels of glucose during AP progression have been reported to influence disease progression by promoting the release of inflammatory cytokines.^10^ The aim of this study was to evaluate the effects of both the presence of DM and the blood glucose level in patients without DM on the severity and course of AP in patients presenting with clinical acute pancreatitis by using computed tomography findings indicating the severity of pancreatitis.

## METHODS

The study included 343 patients who were hospitalized and followed up with the diagnosis of AP in the Gastroenterology Department of Van Training and Research Hospital, Turkey between 2014 and 2018. The diagnosis of AP was made based on the American College of Gastroenterology guidelines.^2^ Those who were allergic to contrast material, those with chronic pancreatitis, those who did not want to participate in the study, and pregnant women were excluded.

### Ethical approval:

All the patients provided written informed consent to participate in the study. Approval for the study was obtained from the Ethics Committee of our hospital (Van, Turkey). (2021/22-08/12/2021, dated February 28, 2017).

### Serum Glucose Level Measurement and Diagnosis of DM:

The diagnosis of DM was made based on accepted clinical criteria. In the previous tests, DM was accepted as pre-prandial plasma glucose > 126 mg / dL (7.0 mmol / L), or during oral glucose tolerance test (carried out with a glucose load containing the equivalent of 75 grams of anhydrous glucose dissolved in water) two hour plasma glucose > 200 mg / dL (11.1 mmol / L), or HbA1C > 6.5% (48 mmol / mol), or random plasma glucose levels in a patient with hyperglycemia or classic signs of hyperglycemic crisis > 200 mg / dL (11.1 mmol / L).^11^

### Acute pancreatitis severity:

All patients included in the study underwent abdominal computed tomography (CT) and pancreatic necrosis and peripancreatic fluid accumulation, ascites, pleural fluid, extrapancreatic parenchymal abnormalities (subcapsular fluid accumulation, hemorrhage or infarction), vascular complications (arterial hemorrhage, venous thrombosis or pseudoaneurysm) and extrapancreatic findings such as gastrointestinal tract involvement (inflammation, intramural fluid accumulation or perforation) were evaluated. Acute pancreatitis was graded and classified according to the Modified Balthazar classification. According to the Balthazar score, patients with scores of 0-2, 4-6 and 8-10 were classified as mild, moderate and severe AP, respectively.

### Statistical Analysis:

Data obtained in the study were analyzed statistically using Statistical Package for the Social Sciences vn. 22.0 software (SPSS, IBM Corpn., Armonk, NY, USA). Categorical data were given as frequency and percentage, and continuous variables as mean and standard deviation values. The parametric data of the groups were compared using the Independent Samples t-test and non-parametric data were compared using the Mann–Whitney U-test. A value of p < 0.05 was considered statistically significant.

## RESULTS

Evaluation was made of 343 patients followed up with the diagnosis of AP, comprising 142 (41.3%) males and 201 (58.7%) females with a mean age of 54.1±17.9 years (age range: 18-97 years). The DM group included 52 (15.1%) patients, and the non-DM group included 291 (84.9%) patients. In the non-DM group, the serum glucose level was <125 mg/dl in 160 (54.9 %) patients, and >125 mg/dl in 131 (45.1%) patients. In the comparisons between the groups in terms of demographic characteristics, the mean age was 57.9±15.5 years in the DM group, and 54.1±17.9 years in the non-DM group, with no statistically significant difference determined between the groups (p>0.05).

The rate of severe AP was significantly higher in the diabetic group according to the Modified Balthazar classification, evaluated on tomographies taken on admission and on the third day (p:0.012, p:0.001, respectively). In the comparison of changes in the severity of the disease (regression, progression, or no-change) from the time of admission to later time-points, the rate of patients with progression was found to be significantly higher in the diabetic group (p:0.031) (Table-I). In the examination made according to the glucose level (125 mg/dl), in the non-DM group, the mild AP rate was significantly lower and the severe AP rate was significantly higher in the group with glucose level >125 mg/dl (p:0.006 and p:0.008, respectively) according to the Modified Balthazar classification, evaluated on tomographies taken at the time of admission. In respect of changes in disease severity (regression, progression, or no-change) from the time of admission to later time-points, the rate of patients with progression was found to be significantly higher in the group with glucose level >125 mg/dl (p:0.013) (Table-II).

**Table-I:** Relationships between the presence of Diabetes Mellitus and Balthazar classification.

	DM n: 52 (15.1%)	Non-DM n:291(84.9%)	Total n:343	P value
***Age (years)***				0.183
Mean, SD	57.9±15.5	54.1±17.9	54.1±17.9	
Range	(28-97)	(18-92)	(18-97)	
***Sex***				0.172
-Male	26 (50.0%)	116 (39.9%)	142 (41.3%)	
-Female	26 (50.0%)	175 (60.1%)	201 (58.7%)	
***Balthazar Classification (on admission)***				0.039*
-Mild	38 (73.1%)	210 (72.2%)	248 (72.3%)	0.892
-Moderate	12 (23.1%)	80 (27.5%)	92 (26.8%)	0.508
-Severe	2 (3.8%)	1 (0.3%)	3 (0.9%)	0.012*
***Balthazar Classification (after 72 hours)***				0.035*
-Mild	36 (69.2%)	214 (73.5%)	250 (72.9%)	0.520
-Moderate	9 (17.3%)	64 (22.0%)	73 (21.2%)	0.447
-Severe	7 (13.5%)	13 (4.5%)	20 (5.9%)	0.011*
***CT changes***				0.025*
-Regression	14 (26.9%)	61 (21.0%)	75 (21.8%)	0.338
-Progression	18 (34.6%)	61 (21.0%)	79 (23.1%)	0.031*
-No change	20 (38.5%)	169 (58.0%)	189 (55.1%)	0.009*
Length of stay in Hospital (days)	6.88±7.4	5.46±5.5	5.12±5.8	0.049*
***Mortality***				
-Yes	50 (96.2%)	288 (99%)	338 (98.5%)	0.119
-No	2 (3.8%)	3 (1%)	5 (1.5%)	

**Table-II:** Relationships between serum glucose level and Balthazar classification in non-diabetic patients.

	Glucose<125 mg/dl N:160(54.9%)	Glucose >125 mg/dl N:131(45.1%)	Total N:291	P value
***Age (years)***				0.324
Mean, SD	51.3±18.6	53.4±15.9	52.8±17.9	
Range	(18-89)	(23-92)	(18-92)	
***Sex***				0.209
-Male	69 (43.1%)	47 (35.9%)	116 (39.9%)	
-Female	91 (56.9%)	84 (64.1%)	175 (60.1%)	
***Balthazar Classification (on admission)***				0.015*
-Mild	126 (78.8%)	84 (64.1%)	210 (72.2%)	0.006*
-Moderate	34 (21.2%)	46 (35.1%)	80 (27.5%)	0.008*
-Severe	0 (0%)	1 (0.8%)	1 (0.3%)	**0.268**
***Balthazar Classification (after 72 hours)***				<0.001*
-Mild	133 (83.1%)	81 (61.8%)	214 (73.5%)	<0.001*
-Moderate	25 (15.6%)	39 (29.8%)	64 (22.0%)	0.004*
-Severe	2 (1.3%)	11 (8.4%)	13 (4.5%)	0.003*
***CT changes***				0.045*
-Regression	35 (21.9%)	26 (19.8%)	61 (21.0%)	0.672
-Progression	27 (16.8%)	38 (29.1%)	65 (22.3%)	0.083
-No change	98 (61.3%)	67 (51.1%)	165 (56.7%)	0.013*
Length of stay in Hospital (days)	4.59±2.8	6.52±7.5	5.01±5.5	0.003*
***Mortality***				
-Yes	160 (100%)	128 (97.7%)	288 (99%)	0.05*
-No	0 (0%)	3 (2.3%)	3 (1%)	

The course of AP severity in both groups was analyzed in detail. In the diabetic group, while there was no patient with Stage-A, it was observed that 2 (33.3%) of 6 patients with Stage-B remained at the same stage, and 4 (66.6%) of regressed to Stage-A on the imaging taken after 72 hours. For further details seeTable-III.

**Table-III:** Evaluation of the changes on the CT scans performed on the 3^rd^ - 7^th^ day compared to the scan performed within the first 12 hours in patient with diabetes mellitus.

CT After 72 hours	CT on admission					

	Stage-A	Stage-B	Stage-C	Stage-D	Stage-E	Total

	n (%)	n (%)	n (%)	n (%)	n (%)	n (%)
Stage-A	0 (0%)	2 (33.3%)	5 (14.7%)	2 (25.0%)	1 (25.0%)	10 (19.2%)
Stage-B	0 (0%)	2 (33.3%)	4 (11.8%)	0 (0%)	0 (0%)	6 (11.5%)
Stage-C	0 (0%)	2 (33.3%)	15 (44.1%)	0 (0%)	1 (25.0%)	18 (34.6%)
Stage-D	0 (0%)	0 (0%)	5 (14.7%)	1 (12.5%)	0 (0%)	6 (11.5%)
Stage-E	0 (0%)	0 (0%)	5 (14.7%)	5 (62.5%)	2 (50.0%)	12 (23.2%)

Total	0 (100%)	6 (100%)	34 (100%)	8 (100%)	4 (100%)	52 (100%)

In the non-DM group, it was observed that 48 (77.4%) of 62 patients with Stage-A remained at the same stage, and 14 (32.6%) showed progression. Of 32 patients with Stage-B, 20 (62.5%) remained at the same stage, 4 (12.5%) regressed to Stage-A, and 8 (25%) progressed to Stage-C on the images taken after 72 hours. For further details see Table-IV.

**Table-IV:** Evaluation of the changes on the CT scans performed on the 3 th-7th day compared to the scan performed within the first 12 hours in patient without diabetes mellitus.

CT After 72 hours	CT on admission					

	Stage-A	Stage-B	Stage-C	Stage-D	Stage-E	Total

	n (%)	n (%)	n (%)	n (%)	n (%)	n (%)
Stage-A	48 (77.4%)	4 (12.5%)	18 (15.5%)	9 (23.7%)	1 (2.3%)	80 (27.5%)
Stage-B	4 (6.5%)	20 (62.5%)	10 (8.6%)	1 (2.6%)	1 (2.3%)	36 (12.4%)
Stage-C	8 (12.9%)	8 (25%)	68 (58.6%)	9 (23.7%)	5 (11.6%)	98 (33.7%)
Stage-D	2 (3.2%)	0 (0%)	12 (10.3%)	11 (28.9%)	3 (7.0%)	28 (9.6%)
Stage-E	0 (0%)	0 (0%)	8 (7.0%)	8 (21.1%)	33 (76.8%)	49 (16.8%)

Total	62 (100%)	32 (100%)	116 (100%)	38 (100%)	43 (100%)	291 (100%)

## DISCUSSION

In our study, the relationship between hyperglycemia and clinical outcomes of acute pancreatitis in DM and non-DM groups was investigated. In the comparison of the severity of AP in the diabetic and non-diabetic groups, it was observed that the rate of severe AP was significantly higher in the diabetic group according to the Modified Balthazar classification, which was made by evaluating computed tomographs taken at the time of admission and after the third day. In addition, the rate of patients with progression in the diabetic group was found to be significantly higher (p:0.035). Similarly, when the non-DM group was evaluated as two groups according to serum glucose level >125 mg/dl or <125 mg/dl, it was seen that severe pancreatitis was significantly higher in the group with high blood sugar levels, and the rate of those who progressed was found to be significantly higher in that same group (p<0.05).

It is not clear why diabetes has such an impact on the severity of AP patients. Some studies on sepsis and critically ill patients may guide why the disease is severe in AP patients with diabetes.^12^,^13^ In these previous studies, the development of multi-organ failure has shown that a high glucose level is a poor prognostic factor in terms of intensive care transfer rate and mortality.^14^ Sun et al. determined that the serum glucose levels and APACHE II scores at the time of presentation were significantly higher in the patient group with pancreatitis, especially in the patient groups with moderate and severe pancreatitis compared to the mild pancreatitis patient group, with evaluations made from the glucose, APACHE II score, TNF-α and CRP values measured at the time of admission.^15^ In a study in Taiwan of 18,990 diabetic patients with a first AP attack and 37,980 non-diabetic patients, it was shown that the rate of severe AP attack was significantly higher in the diabetic group (adjusted odds ratio [OR] 1.21, 95%CI: 1.16–1.26, p<0.001).^16^ It has long been known that high glucose levels cause oxidative stress in various tissues of the body. Chronic hyperglycemia has been shown to increase mitochondrial oxidative stress through reactive oxygen production and lipid peroxidation.^17^ Elevated glucose has also been shown to activate pancreatic stellate cells via protein kinase C and p38 mitogen-activated protein kinase.^18^ Therefore, the glucose level is closely related to inflammatory responses in AP and may affect disease progression

These data suggest that even acute changes in blood glucose are important in the progression and severity of the disease. In a retrospective cohort study of 4,946 critically ill patients by Egi et al., the mortality rate was seen to be 1.74-fold higher in non-diabetic patients than diabetic patients (n=728) in the intensive care unit with the same glucose concentration(144-180 mg/dl) range.^19^ Similarly, a meta-analysis investigating the effect of glycemic modification in critically ill patients (n = 162,259) found a consistent association between glycemic modification and short-term mortality.^20^ These findings suggest that acute and chronic hyperglycemia act through different pathophysiological pathways and therefore may lead to different clinical outcomes.^21^

### Limitations:

This was primarily the retrospective study and risk factors that play a role in disease progression were not discussed in detail. In addition, since the data were obtained from only one tertiary-level centre, they may not include all patients hospitalized with a diagnosis of AP. However, the strengths of the study were the large sample size, including diabetic and non-diabetic patient groups, and the clear demonstration of the severity and course of the pancreatitis according to the Balthazar classification, and evaluation of all CT scans by the same radiology specialist.

## CONCLUSION

Knowing the course of the disease at the time of admission is important for starting the optimum treatment and close follow-up of the patient. Elevated blood sugar is relatively common in patients with AP and has a negative impact on the disease process. A high glucose level can increase the severity of AP and slow down the healing process. It can be considered that the presence of hyperglycemia provides information about the course of the disease since there is a correlation between the glucose value measured at the beginning and the severity of the disease according to CT taken later.

### Authors Contribution:

**GK**, **GEK** and **YK:** Conceived, designed and did statistical analysis & editing of manuscript, is responsible for integrity of research.

**AO**, **GK** & **YK:** Did data collection, review and manuscript writing.

**GK** and **YK:** Did review, was involved in literature searsch and final approval of manuscript.
